# Exploration of utilizing electronic health databases in evidence-based practice among Iranian pharmacists: a survey by simulation of health-seeking pregnant women

**DOI:** 10.1186/s40780-026-00561-7

**Published:** 2026-04-02

**Authors:** Hooman Pourbala, Ghader Mohammadnezhad, Hadi Esmaily

**Affiliations:** 1 Department of Pharmacology and Toxicology, School of Pharmacy, Shahid Beheshti University of Medical Sciences, Tehran, Iran; 2 Student Research Committee, School of Pharmacy, Shahid Beheshti University of Medical Sciences, Tehran, Iran; 3 Department of Clinical Pharmacy, School of Pharmacy, Shahid Beheshti University of Medical Sciences, 3rd Floor, 2660th Valiasr Street, Niyayesh Intersection, Tehran, Iran

**Keywords:** Evidence-based medicine, Public health, Pharmacy practice, Health services research

## Abstract

**Background:**

Evidence-based medicine (EBM) applies the most reliable evidence available to support clinical decision-making, improving treatment outcomes, rational medication use, and reducing healthcare costs.

**Objectives:**

This study evaluated the knowledge, attitude, and practice (KAP) of Iranian community pharmacists regarding EBM utilization.

**Methods:**

A 36-item questionnaire was designed and validated to assess the pharmacists’ knowledge and attitudes toward EBM. Practice was evaluated using a simulated-patient method one week after questionnaire completion. Multivariable linear regression was used to examine associations between pharmacist characteristics and knowledge and practice scores, with sensitivity analyses adjusting for geographic region.

**Results:**

Within 284 pharmacy visits, data were obtained from 200 pharmacists aged 24–65 years. The average knowledge score was 37.91 ± 15.4 (out of 100). Pharmacists aged 24–29 demonstrated higher knowledge levels than those aged 30–65 (*P* = 0.042). Dispensing more than 30 prescriptions daily was associated with higher knowledge scores (*P* = 0.024). Pharmacists who reported using UpToDate^®^ demonstrated higher knowledge and practice scores compared with non-users (*P* < 0.001). Attitudes toward EBM were overwhelmingly positive, indicating strong theoretical support despite reported implementation barriers. The mean practice score in managing the simulated-patient was 50.34 ± 21.35. A direct association was detected between knowledge and practice (*r* = 0.73, *P* < 0.05), whereas no association was observed between attitudes and practice. Multivariable analysis showed that younger age, higher prescription volume, and use of UpToDate^®^ were independently associated with higher knowledge and practice scores.

**Conclusion:**

Most pharmacists support EBM; however gaps in knowledge and practice remain. Use of structured electronic resources such as UpToDate^®^ were associated with improved knowledge and practice.

**Supplementary Information:**

The online version contains supplementary material available at 10.1186/s40780-026-00561-7.

## Introduction

Pharmacists play a pivotal role in enhancing public health outcomes, including the management of chronic diseases and health promotion. In community pharmacies, they uphold ethical and professional obligations by advising patients on medication usage and managing common minor ailments, such as headaches, colds, and allergies, through the provision of over-the-counter (OTC) treatments [1–3]. To effectively fulfill these responsibilities, pharmacists must be well-versed in the principles of evidence-based medicine (EBM), which is defined as the systematic and judicious use of the most current and reliable evidence to inform clinical decisions regarding individual patient care [2, 4, 5].

Prominent organizations, including the World Health Organization (WHO), the American Association of Colleges of Pharmacy (AACP), and the International Pharmaceutical Federation (FIP), have emphasized the necessity of integrating EBM into pharmacy practice to ensure good pharmacy practice (GPP) [6–8]. By employing EBM, pharmacists can significantly enhance the effectiveness of treatment plans, thereby improving patient outcomes and minimizing unnecessary interventions [9, 10]. This approach not only fosters patient adherence to prescribed therapies but also promotes the optimal use of medications and the efficient allocation of clinical resources within the community. Ultimately, the adoption of EBM is associated with improved quality of life for patients and a reduction in the socioeconomic burden of diseases [11–13].

One effective strategy for evaluating the application of EBM in practice is the simulated patient method (SPM). This method serves as a valuable tool to accurately assess healthcare professionals’ practices. A simulated patient (SP) is trained to authentically represent a patient, consistently presenting a specific chief complaint [14–16]. However, the SPM has not been widely adopted in pharmacy practice in Iran. By employing the SPM, researchers can assess real-life scenarios rather than relying solely on self-reported performance, thus identifying strengths and weaknesses in pharmacists’ interactions with patients [17, 18]. This method provides diverse scenarios that evaluate a broader range of pharmacists’ skills and knowledge, yielding insights into the professionalism and clinical competence of healthcare providers. Consequently, these insights can inform the development of targeted strategies to address identified performance deficiencies. Designing an effective SP scenario poses several challenges, as it requires considerable time and effort to understand the specific needs of the target population. It is essential to maintain flexibility in the framework to adapt to unforeseen changes [16, 19, 20].

Various e-health platforms, including Cochrane^®^, Medscape^®^, Micromedex^®^, and UpToDate^®^ are available for seeking health information. However, previous studies have indicated that these tools are not widely integrated into the daily practices of healthcare professionals, leaving them reliant on traditional methods for information retrieval [21, 22]. This research aimed to assess the knowledge, attitudes, and practices (KAP) of Iranian pharmacists regarding the utilization of electronic information databases regarding the implementation of EBM in community.

## Methods

### Study design & setting

A cross-sectional survey using a mixed-methods approach was conducted from December 2021 to December 2022 in Tehran, Iran, which comprised two components: a researcher-designed questionnaire to assess the knowledge and attitude of participants, and an SPM for evaluating their practices. The manuscript followed the Strengthening the Reporting of Observational Studies in Epidemiology (STROBE) guidelines for standardization [23].

### Ethical consideration

The Ethics Committee of Shahid Beheshti University of Medical Sciences approved the study protocol with the registered code IR.SBMU.PHARMACY.REC.1399.212. The study objectives and procedures were explained to the pharmacists to help their full comprehension. Furthermore, a written informed consent was obtained from the pharmacists to convey the purpose, potential risks, and benefits of the study. The main reason for acquiring verbal informed consent was to conceal the SP during regular visits of the patients. The Ethics Committee of Shahid Beheshti University of Medical Sciences approved the procedure for obtaining verbal informed consent.

### Participants

Community pharmacists (CPs) of Tehran were invited to participate in this survey. The study utilized Health Information Exchange (HIX), an online national pharmacy management system in Iran (http://pharmacy.fda.gov.ir), which maintains records of all pharmacies, including their addresses, details about the pharmacists employed, and contact numbers. At the time of the study, a query from this system indicated that there were approximately 2,500 pharmacies operating in Tehran and the sample size was calculated based on this information. During an initial meeting with the CPs, the research team outlined the study’s objectives, emphasized ethical considerations, and highlighted the importance of maintaining participant confidentiality. Regarding the eligibility criteria, participants were included if they provided written informed consent to complete the questionnaire and engage with the SP. Pharmacists who did not complete the questionnaire were excluded from the study.

### Data sources & variables

We collected demographic information from the participants during the initial meeting. This information included their age, gender, highest level of education, work experience, average hours worked per day in the pharmacy, years since graduation, the usual number of daily prescriptions checked, and regular use of scientific resources and references. Furthermore, the measured variables by the questionnaire were knowledge and attitude of CPs toward EBM. The questionnaire assessed multiple predefined information-seeking modalities, including general search engines (e.g., Google), medical databases (e.g., Medscape^®^), consultation with colleagues, drug information centers, and point-of-care clinical decision support systems such as UpToDate^®^ (Supplementary File 1). In addition, we informed participants that the data collection process would be finalized within a week, utilizing an SPM for practice measurement.

### Outcome measurements

To achieve the study outcomes, we implemented the following methodologies:

#### Development of the knowledge & attitude questionnaire

Initially, an expert panel comprised ten specialists from various fields, including clinical pharmacy, pharmacy practice, family medicine, nutritional science, pharmacology, and toxicology. The expert panel referred to the university faculty members who collaborated to consult and design the case scenario. A questionnaire was designed based on six essential domains identified by the panel to assess the knowledge and attitude of healthcare professionals towards EBM. These domains were the knowledge of EBM principles, familiarity with EBM resources, attitude toward EBM, application of EBM in practice, barriers to EBM implementation, continuing education and training needs.

The questionnaire was developed following a thorough review of literature and underwent three rounds of editing. After revising the questionnaire, the content validity of it was evaluated regarding simplicity, clarity, relevance, and necessity. Content validity index (CVI) and content validity ratio (CVR) were calculated based on expert opinions. A CVI greater than 0.8 was deemed acceptable according to Waltz and Baussel’s criteria [24].

To assess the internal consistency of the questionnaire using Cronbach’s alpha coefficient, we provided it to 50 pharmacists during the Iranian Clinical Pharmacy Congress. Upon the collection of the responses, an alpha value of 0.7–0.9 was considered as an acceptable range of internal consistency. The results of these 50 volunteers were not included in the final analysis. The questionnaire was translated following standard guidelines for translation, which involve preparation, forward translation/matching, back translation done by an authorized translator, harmonization, and validation to publish it in English. Final version of the knowledge and attitude questionnaire is available in the Supplementary File 1.

#### Assessing the pharmacy practice by a simulated patient

The SP scenario was developed according to the Standards of Best Practice for Simulation (SOBP) [20]. The SOBP consists of five main domains: (1) Safe work environment: Three crucial principles were taken into account to foster respect, ensure adherence to safe work practices, and maintain confidentiality. (2) Case Development: Two fundamental principles guide the development of SP case materials: thorough preparation and consideration of case components. Expertise in creating teaching and assessment materials is crucial for SP educators, considering the critical role that case-related materials play in their work. (3) Training SPs: Comprehensive training is essential for SPs as it equips them with the necessary skills to accurately and consistently portray roles, provide effective feedback, and proficiently utilize assessment tools. SP educators have to integrate and align the development of these individual skills with the SP’s unique experience and the educational objectives of the practice. (4) Program management: SP programs should be cost-effective and adhere to purpose, expertise, policies, procedures, precise records control, teamwork, and quality. (5) Professional development: SP educators actively engage in professional development to foster sufficient expertise in clinical practices.

To evaluate the feasibility of the SPM and identify any weaknesses, a preliminary pilot study was conducted with 50 pharmacists in Tehran. The results of these 50 pharmacists were not included in the final analysis. The approach of SP in pharmacy practice evaluation is detailed in the Supplementary File 1. Each response from the pharmacist and the steps they take during the history-taking process with the SP served as key criteria for assessing the professional and clinical competence of the participants.

To prevent the SP from misleading the study participants, the names and profiles of the pharmacists were received in the questionnaire. Subsequently, the pharmacists’ pictures were obtained from the Iran’s medical system database (https://membersearch.irimc.org/) and provided them to the SP. This allowed the SP to refer to the desired pharmacist according to the picture. If the pharmacist was not present in the pharmacy, that pharmacy would be visited again.

##### Case scenario

**Fig. 1:**
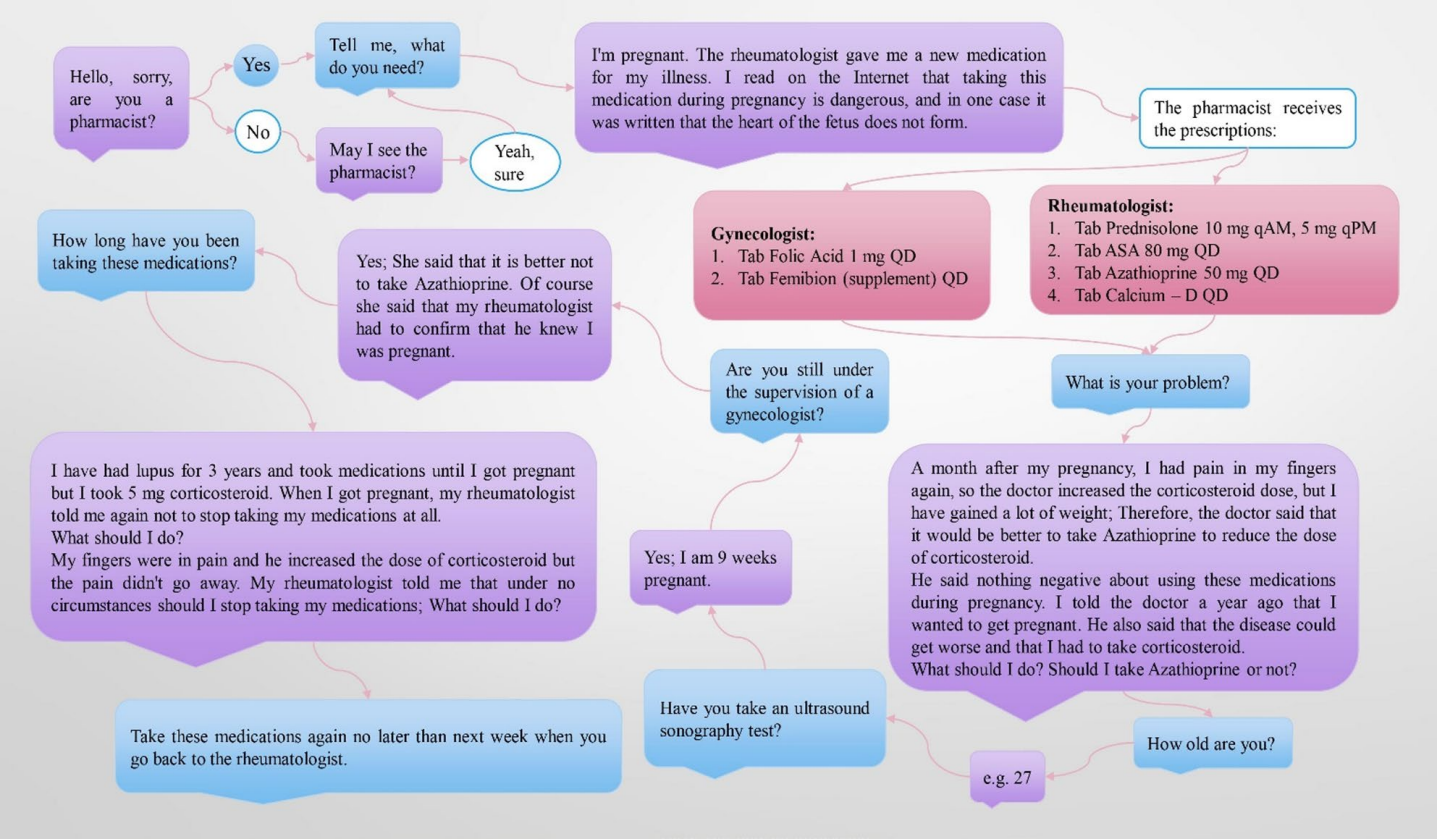
A hypothetical case scenario scheme

A female pharmacy student was chosen and given specialized training to act as an SP whose physical attributes closely resembled a pregnant woman’s. We provided education to our student regarding a 25–35 years old woman with a medical history of systemic lupus erythematosus. She was uncertain about taking a particular prescription medication during her ninth week of pregnancy. Figure 1. represents an overview and examples of potential responses a pharmacist might give to the SP.

### Scoring systems

The quality of medication counseling was assessed based on two key domains: [1] logical questioning to assess patient needs and [2] providing accurate advice with clear instructions for medication use. An inductive approach was used to develop a comprehensive scoring criteria set focused on these domains [25].

The scoring system was designed by the expert panel for this project. A general core was assumed for classifying the pharmacist’s questions, recommendations, and overall clinical approach to the case. As a result, a binary scoring system (fulfillment/non-fulfillment) was created for each predefined item, as detailed in Supplementary File 2. Using this framework, the knowledge score was calculated as an expert-weighted composite index encompassing multiple domains of evidence appraisal and information literacy, yielding a standardized total score ranging from 0 to 100. Similarly, the practice score was derived from a structured SP checklist incorporating weighted binary (yes/no) and graded items with predefined coefficients and was subsequently transformed into a standardized composite score (0-100). For implementation, the SP evaluated each encounter immediately upon exiting the pharmacy using this standardized checklist. The final practice score was automatically calculated from these responses to ensure objectivity and minimize rater bias.

### Bias

We only invited individuals who fully met the eligibility criteria to address the selection bias. As knowledge and attitude have been assessed using scientific questions, we tend to reduce the risk of social desirability bias, which could occur if pharmacists altered their responses based on perceived expectations. In addition, the pharmacists were not informed of details regarding the SP’s identity, medical condition, or the specific timing of the visit. This lack of information was crucial in helping an unbiased assessment of their actual practice.

Considering that pharmacies in Tehran serve an average of around 250 clients per day, and since the SP visit happened several days after the initial visit, the potential for recall bias was deemed minimal. To further address potential biases associated with the SP, she was rigorously trained to adhere strictly to a standardized script during visits, ensuring consistency in the interactions with pharmacists. By following this protocol, we aimed to closely replicate typical practice conditions, thereby reducing the likelihood of behavior modification resulting from the awareness of being observed. Furthermore, the scenario provided by the SP was not directly connected to the knowledge and attitude questionnaire, which diminished the possibility of bias.

### Sampling

A cluster-stratified sampling approach was implemented to recruit community pharmacies from all five major geographic and socio-economic regions of Tehran (northern, southern, eastern, western, and central areas). Pharmacies within each stratified cluster were then selected based on convenience to achieve this primary goal of geographic diversity. While this ensured a representative spread across the city, the use of convenience sampling within clusters may affect the CPs’ representativeness. The sample size was calculated using Cochran’s statistical formula, tailored to the study population. Our objective was to achieve a precision level of 7% within a 95% confidence interval, based on a target population of about 2500 pharmacies. Considering that each pharmacy is associated with a single pharmacist in Iran, we determined that a minimum sample size of 182 pharmacists was necessary to meet this precision requirement.

### Data analysis

Data analysis was conducted using SPSS version 26 software. Descriptive statistics were reported as frequencies and means ± standard deviation (SD). The Kolmogorov-Smirnov test was employed to assess the normality of the data distribution. Given their composite structure, wide score range, and approximate normal distribution, the knowledge and practice scores were treated as continuous variables for statistical analyses. To compare the sub-groups that existed in the characteristics of the participants, both the t-test and analysis of variance (ANOVA) were utilized. The Chi-Square test was applied to examine relationships between non-parametric variables. Additionally, Tukey’s Honestly Significant Difference (HSD) post-hoc test was conducted for multiple comparisons following ANOVA. The relationship between the average knowledge and performance scores of participants and the sub-groups of their characteristics was also assessed using the Chi-Square test. Multivariable linear regression was used to identify factors independently associated with knowledge and practice scores. The initial models included the following covariates, selected a priori: age, years of experience, number of daily prescriptions dispensed, and use of clinical resources. To assess the robustness of our findings against the stratified sampling design, we performed a sensitivity analysis by adding the geographic region (a 5-level categorical variable) as an additional covariate. The attitude items were conceptualized as reflecting distinct, multidimensional facets of belief (e.g., theoretical value of EBM versus practical barriers to its implementation) rather than a single unidimensional construct. Consequently, internal consistency was assessed using Cronbach’s alpha, and because reliability was low, these items were analyzed and reported individually to preserve the interpretive nuance of each dimension. A significance threshold of *P* < 0.05 was set to determine statistical significance.

## Results

**Fig. 2:**
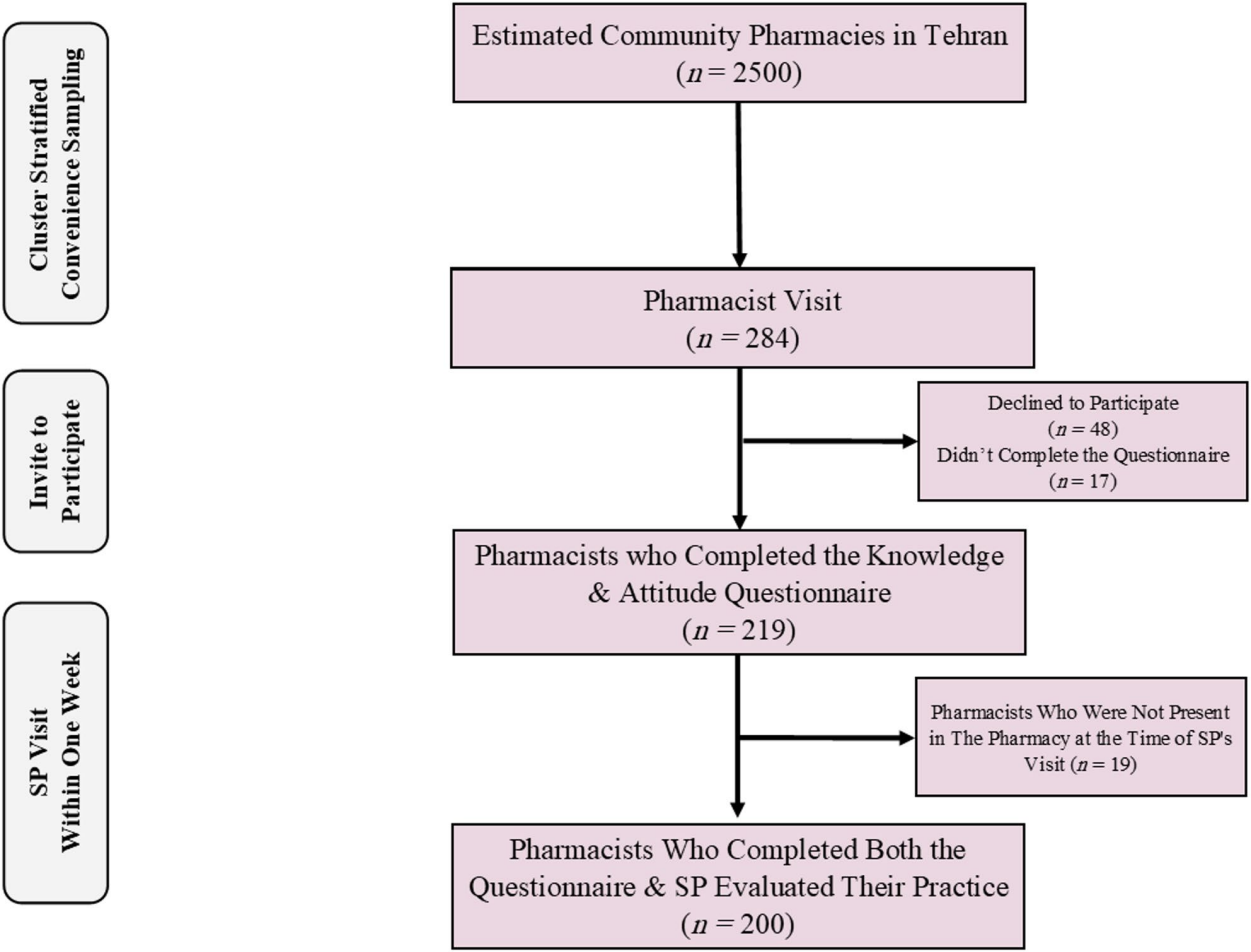
Flow diagram of study participant recruitment and inclusion. Note: The sampling frame comprised an estimated 2500 community pharmacies in Tehran. In accordance with national regulations, each pharmacy is managed by one pharmacist per shift, making this a proxy for the available pharmacist population. Subsequent boxes detail the flow of individual participants through the study stages

The prototype questionnaire achieved a CVI of 0.82 and a Cronbach’s alpha of 0.81 for 50 pharmacists. Within 284 pharmacy visits, 200 pharmacists completed the study per protocol. After a week, their practice was evaluated with the SP. Of the 84 participants excluded from the analysis, 48 decided not to participate, 17 did not finish the questionnaires, and 19 pharmacists were not available during the SP’s visit. Figure 2. shows the flow diagram of participants in more detail.

Out of 200 included pharmacists, 68 were men, and 132 were women. Regarding the highest level of education, 92.5% had a PharmD, 6% had a PhD, and 1.5% had a B.Pharm. Detailed demographic data of the study participants can be found in Table 1.

**Table 1:** Demographic characteristics of pharmacists (*N* = 200)

Variables	Subgroups	*N* (%)
Age	20–29	60 (30)
	30–39	67 (33.5)
	40–49	38 (19)
	50–65	35 (17.5)
Gender	Male	68 (34)
	Female	132 (66)
Highest level of education	Pharm.D.	185 (92.5)
	Ph.D.	12 (6)
	B.Pharm.	3 (1.5)
Work experience	Less than 5 years	79 (39.5)
	5–10 years	52 (26)
	More than 10 years	69 (34.5)
Years since graduation	1–10 years	94 (47)
	11–20 years	54 (27)
	21–30 years	29 (14.5)
	More than 30 years	23 (11.5)
Use of scientific resources and references	Online resources	161 (80.5)
	Electronic information databases (UpToDate^®^)	95 (47.5)

Table 2. shows the results of the pharmacists’ attitudes about the impact of utilizing electronic information databases. Analysis of these attitudes revealed a multidimensional structure. As mentioned in the Methods, the attitude items demonstrated low internal consistency as a scale (Cronbach’s α = 0.350), confirming they represent separate attitudinal constructs. The results are therefore presented thematically below, revealing a clear pattern: strong theoretical endorsement of EBM principles (Q6, Q7, Q9) coexisting with significant concerns about practical barriers to its application (Q1, Q3, Q4, Q8). Many pharmacists (58%) believed that the references available in community pharmacies were inadequate for answering the patients’ questions (Q1). Most pharmacists (95.5%) do not believe that having a lot of work experience and personal knowledge improves the quality of pharmacy practice (Q2). Most pharmacists (86.5%) believed that it becomes impractical to use medical resources to help clients when pharmacies are overcrowded (Q3). In addition, a majority of 65.5% believed that searching for information in scientific resources in the presence of the patient can cause cynicism among patients, while 22% had the opposite opinion (Q4). Likewise, 62.5% of pharmacists disagreed that a pharmacist’s work experience is more efficient than searching with scientific resources. Conversely, 18.5% of pharmacists agreed with this statement (Q5). Pharmacists indicated it is important to consult reliable scientific resources to ensure the safe use of drugs during pregnancy and breastfeeding. They also agreed on the importance of pharmacists checking prescription drug interactions in community pharmacies (Q6, Q7). Most participants (71.5%) disagreed with the idea that guidance, counseling, and the use of credible scientific resources in pharmacies cause confusion and anxiety in patients (Q8). Most pharmacists (78.5%) believed that using scientific resources and EBM improves patients’ satisfaction and adherence to pharmacotherapy (Q9). The overall pattern reveals a pronounced positive skew in the attitude data, indicating strong theoretical agreement with the value of EBM. This consensus and lack of variability precludes meaningful correlation analysis but underscores a systemic theory-practice gap.

**Table 2:** The attitudes of pharmacists towards health services and EBM in community pharmacies

1. Pharmacists’ attitudes towards using references to answer patients’ questions.	Agree	83 (41.5%)
	Disagree	116 (58%)
	Neutral	1 (0.5%)
2. Pharmacists’ attitudes towards their knowledge and years of experience.	Agree	6 (3%)
	Disagree	191 (95.5%)
	Neutral	3 (1.5%)
3. Pharmacists’ attitudes towards the impossibility of searching for answer patients’ questions during the busy time of the pharmacy.	Agree	173 (86.5%)
	Disagree	23 (11.5%)
	Neutral	4 (2%)
4. Pharmacists’ attitudes towards patients’ negative mentality towards pharmacist knowledge when referring to scientific resources.	Agree	131 (65.5%)
	Disagree	44 (22%)
	Neutral	25 (12.5%)
5. Pharmacists’ attitudes towards the effectiveness of work experience instead of referring to scientific resources.	Agree	37 (18.5%)
	Disagree	125 (62.5%)
	Neutral	38 (19%)
6. Pharmacists’ attitudes towards the need to use scientific resources to check the risks of medicines during pregnancy and breastfeeding for all patients.	Agree	199 (99.5%)
	Disagree	0
	Neutral	1 (0.5%)
7. Pharmacists’ attitudes regarding the non-necessity of checking drug interactions by a pharmacist versus the examination of drug interactions by a physician.	Agree	0
	Disagree	200 (100%)
	Neutral	0
8. Pharmacists’ attitudes regarding anxiety and confusion in managing patients will change and reduce the acceptance of medications if the information is provided completely.	Agree	21 (10.5%)
	Disagree	143 (71.5%)
	Neutral	36 (18%)
9. Pharmacists’ attitudes towards increasing patient satisfaction by using reliable resources to respond to patients.	Agree	157 (78.5%)
	Disagree	8 (4%)
	Neutral	35 (17.5%)

The results in Table 3. indicate that many pharmacists did not utilize a suitable scientific resource and underperformed in obtaining the patient’s medical history and making a proper clinical decision. Altogether, their practice was poor to moderate. The study found that pharmacists’ practice scores ranged from 13 to 90 out of 100, averaging 50.34 ± 21.35.

**Table 3:** Pharmacists’ practice towards EBM implementation and taking past medical history

1. Pharmacist statement about clinical management after taking a complete history.	Yes	131 (65.5%)
	No	69 (34.5%)
2. Ask the SP’s age.	Yes	24 (12%)
	No	176 (88%)
3. Ask for past medical history.	Yes	113 (56.5%)
	No	87 (43.5%)
4. Writing notes while taking history.	Yes	32 (16%)
	No	168 (84%)
5. Ask for family health history.	Yes	41 (20.5%)
	No	159 (79.5%)
6. Ask about taking medications.	Yes	140 (70%)
	No	60 (30%)
7. Ask about a history of drug or food allergies.	Yes	9 (4.5%)
	No	191 (95.5%)
8. Quick selection and correct use of scientific and information resources.	Yes	59 (29.5%)
	No	141 (70.5%)
9. Provide the proper clinical decision.	Yes	60 (30%)
	No	140 (70%)
10. Professional discipline in managing the SP. (Score)	4	36 (18%)
	3.5	34 (17%)
	3	23 (11.5%)
	2.5	24 (12%)
	2	27 (13.5%)
	1.5	38 (19%)
	1	13 (6.5%)
	0.5	5 (2.5%)
11. Provide needed education in plain and understandable language for the SP. (Score)	3	1 (0.5%)
	2.5	16 (8%)
	2	76 (38%)
	1.5	92 (46%)
	1	14 (7%)
	0.5	2 (1%)
12. Explain follow-up tips to the SP after clinical decision.	Yes	42 (21%)
	No	158 (79%)
13. Receive educational feedback from the SP.	Yes	9 (4.5%)
	No	191 (95.5%)
14. Overall practice assessment.	Very Poor	57 (28.5%)
	Poor	77 (38.5%)
	Good	65 (32.5%)
	Very Good	1 (0.5%)

Abbreviation: SP, Simulated patient

The assessment of pharmacists’ knowledge reveals that their overall score in EBM is 37.91 ± 15.4, signifying a range of weak to moderate proficiency. Table 4. shows the relationship between knowledge scores and other study parameters.

**Table 4:** Relationships between knowledge and practice scores with other variables

Characteristics	Subgroups	Knowledge Score	*P*-value	Practice Score	*P*-value
Age	24–29	44.22 ± 16.11	0.042*	48.2 ± 16.4	0.047*
	30–39	39.80 ± 12.85		53.0 ± 23.2	
	40–49	40.00 ± 14.43		49.5 ± 20.7	
	50–65	37.20 ± 19.11		48.1 ± 21.0	
Gender	Male	37.44 ± 14.28	0.688	50.4 ± 21.0	0.997
	Female	38.39 ± 16.52		50.3 ± 21.7	
Highest level of education	Pharm. D.	38.17 ± 15.72	0.966	50.3 ± 22.0	0.886
	Ph.D.	37.00 ± 17.97		51.7 ± 22.8	
	B. Pharm.	36.00 ± 30.40		45.0 ± 32.1	
Work experience (years)	< 5	37.95 ± 13.63	0.242	46.5 ± 22.4	0.038*
	5–10	35.49 ± 15.02		49.4 ± 21.1	
	> 10	40.15 ± 18.31		55.4 ± 21.8	
Number of daily prescriptions dispensed	< 10	36.35 ± 15.19	0.047*	51.7 ± 21.6	0.07
	10–20	36.60 ± 14.22		45.2 ± 21.3	
	20–30	35.05 ± 13.77		53.1 ± 22.9	
	> 30	46.51 ± 20.64		56.8 ± 21.5	
Use of scientific resources and references	Online resources	35.51 ± 15.33	0.032*	46.2 ± 21.5	0.018*
	Electronic information databases (UpToDate^®^)	39.86 ± 17.00		52.8 ± 22.2	
	Both	45.00 ± 17.86		59.3 ± 21.9	

Note: *Significant at *P* < 0.05

Among the various information-seeking modalities assessed, only the use of UpToDate^®^ showed a statistically significant association with higher knowledge and practice scores. Analysis of the study population revealed that younger and less experienced pharmacists, as well as those who used electronic clinical resources such as UpToDate^®^, showed better practice regarding SP. A direct association was detected between knowledge and practice scores (*r* = 0.73, *P* < 0.05), providing a statistical basis for this observation. It is important to note that these two scores measure related but distinct constructs: the knowledge test assesses factual recall, while the SPM evaluates applied performance, which incorporates additional skills like clinical reasoning and communication. The results revealed that most pharmacists have a limited to moderate understanding of EBM. Subsequently, their clinical practice reflects this insufficient knowledge. There was a positive correlation between the number of prescriptions dispensed daily and the enhancement of pharmacists’ knowledge. However, it did not significantly affect their practice. Subgroup analysis is also shown in detail in Table 4.

### Factors associated with knowledge and practice scores

Multivariable linear regression analyses showed that age, number of daily prescriptions, and use of UpToDate^®^ were independently associated with both knowledge and practice scores (Tables 5 and 6). Professional experience was significantly associated with knowledge, but was not significantly associated with practice (*P* = 0.063).

### Sensitivity analysis with geographic region

Sensitivity analyses incorporating geographic region were conducted to assess the stability of the multivariable models (Tables 5 and 6). For knowledge scores, the direction and statistical significance of associations with age, number of daily prescriptions, professional experience, and use of UpToDate^®^ remained unchanged after inclusion of geographic region, while the corresponding regression coefficients showed only minor variation. For practice scores, associations with younger age, prescription volume, and use of UpToDate^®^ were similarly maintained with minimal changes in effect estimates, whereas professional experience remained not significantly associated with practice. Geographic region was not significantly associated with either outcome.

**Table 5:** Multivariable regression models for knowledge score

Association	Primary Model (Without Region)		Sensitivity Model (With Region)	
	B Coefficient	*P*-value	B Coefficient	*P*-value
Constant	32.387	< 0.001*	33.280	< 0.001*
Age	-5.333	< 0.001*	-5.239	< 0.001*
Experience	2.793	0.015*	2.691	0.019*
Number of Daily Prescriptions	4.760	< 0.001*	4.774	< 0.001*
UpToDate	8.606	< 0.001*	8.889	< 0.001*
Region	-	-	-0.625	0.335

Note: B represents the unstandardized coefficient. *Significant at *P* < 0.05

The primary and sensitivity analysis models for practice scores are presented in Table 6.

**Table 6:** Multivariable regression models for practice score

Association	Primary Model (Without Region)		Sensitivity Model (With Region)	
	B Coefficient	*P*-value	B Coefficient	*P*-value
Constant	50.498	< 0.001*	51.721	< 0.001*
Age	-9.707	< 0.001*	-9.578	< 0.001*
Experience	2.887	0.063	2.747	0.078
Number of Daily Prescriptions	2.446	0.043*	2.465	0.042*
UpToDate	15.142	< 0.001*	15.529	< 0.001*
Region	-	-	-0.856	0.331

Note: B represents the unstandardized coefficient. *Significant at *P* < 0.05

## Discussion

This study represents the first examination of the relationship between e-health literacy and KAP among Iranian CPs. We employed a mixed-methods approach that incorporated two components: a questionnaire designed by an expert panel to assess pharmacists’ knowledge and attitudes, alongside an SPM to evaluate their practices. The novelty of our research is underscored by the critical influence that patient consultations with pharmacists can have on both maternal and fetal health, emphasizing the need for pharmacists to verify updated resources to support EBM. It should be noted that the SPM scenario was specifically designed around a complex, high-risk case to stress-test evidence-based decision-making under the most demanding conditions encountered in real-world settings. However, pregnancy-related counseling involves unique considerations (e.g., maternal-fetal safety) that may not reflect routine EBM applications in other patient populations. Therefore, the findings of our study may not be fully generalizable to all types of pharmacist-patient interactions concerning more common ailments.

Our findings indicate that Iranian pharmacists generally lack sufficient knowledge about available resources, which hinders their ability to search for and study valid and current publications. Furthermore, most pharmacists reported insufficient time as a key obstacle to utilize these resources properly within community pharmacy settings. The overwhelmingly positive attitude toward EBM, while a favorable cultural indicator, demonstrated a ceiling effect within the studied population. This lack of attitudinal variance effectively renders it a constant, thereby shifting the critical research question from whether pharmacists value EBM to why they were unable to operationalize these values in practice. This pivot strongly suggests that the primary impediments are not attitudinal but are instead structural and systemic in nature, such as time constraints, workflow inefficiencies, or lack of access to resources.

The structure of the pharmacists’ attitudes provides a crucial key to understanding the theory-practice gap observed in this study. The low reliability of a hypothetical attitude scale is not a measurement failure but an empirical reflection of a conflicted mindset. Pharmacists simultaneously hold a strong theoretical belief in the value of EBM and a palpable awareness of the contextual barriers that hinder its execution. It is this tension, rather than a simple lack of positive attitude, that appears to underpin the gap between knowledge and practice.

In addition, we found that participants who utilized UpToDate^®^ and online resources demonstrated significantly higher knowledge and practice scores. These findings indicate that engagement with reliable, evidence-synthesizing online resources may be associated with higher levels of professional knowledge and clinical performance among pharmacists. A positive correlation was observed between higher knowledge and better practice among CPs, suggesting a solid factual foundation is an important component of clinical performance. However, since these two methods assess related but distinct constructs, this correlation should be interpreted with caution. Both knowledge and practice scores are composite indices derived from multiple domains and therefore represent approximate rather than exact interval-level measures. The imperfect nature of this relationship highlights that performance in a simulated setting is influenced by factors beyond factual knowledge alone, such as communication skills, confidence, and the ability to apply information under time pressure. Accordingly, knowledge does not uniformly or directly translate into performance across individuals.

Given the cross-sectional design of this study, the observed associations cannot be interpreted as causal. Reverse association is plausible, whereby pharmacists with higher baseline EBM knowledge, greater professional motivation, or stronger engagement with clinical practice may be more inclined to use electronic clinical resources such as UpToDate^®^, rather than resource use necessarily leading to improved knowledge or practice.

The use of evidence-synthesizing electronic resources such as UpToDate^®^ may also facilitate rapid access to reliable information in clinical settings characterized by substantial time constraints. Thus, the associations between UpToDate^®^ use and pharmacists’ knowledge and practice scores can be interpreted from two complementary perspectives: first, that use of such resources may support higher knowledge and better clinical performance; and second, that pharmacists with stronger knowledge and practice may preferentially select UpToDate^®^ as an accessible and efficient information source.

Additionally, our results demonstrated a concerning trend: pharmacists’ knowledge and familiarity with resources for addressing patients’ medication needs tend to decline or remain static as they age. It can be inferred that older pharmacists often face multiple challenges such as inadequate facilities and limited opportunities for ongoing training, which may lead to a gradual decline of their knowledge and skills. However, younger pharmacists not only benefit from advanced scientific knowledge, but also demonstrated greater familiarity with reputable databases and references. In this study, most Iranian pharmacists held a PharmD degree, while others had a bachelor’s degree or a PhD. However, no statistically significant difference was observed in knowledge levels across these educational attainment groups. Also, while the majority of surveyed pharmacists had less than five years of work experience, no correlation was identified between knowledge levels and work experience among pharmacists with < 30 years in the profession compared to other groups. Moreover, younger and less experienced pharmacists demonstrated higher knowledge and proficiency in clinical management.

Our multivariable analysis, controlling for key demographic and practice characteristics, identified factors independently associated with knowledge and practice among CPs. Younger age and UpToDate^®^ utilization emerged as robust factors independently associated with higher knowledge and practice scores, highlighting the potential advantages of more recent training and access to reliable point-of-care resources. Prescription volume was also independently associated with knowledge and practice, suggesting that high-throughput environments may reinforce the application of knowledge and decision-making efficiency, or that more competent pharmacists attract a larger patient load. The stability of these associations was supported by a pre-specified sensitivity analysis adjusting for geographic region, indicating robustness to potential sampling variation. Notably, greater professional experience was associated with higher knowledge but not practice, delineating a theory-practice gap in which accumulated knowledge does not automatically translate into optimal clinical action.

Several studies have explored the impact of age and experience on EBM among healthcare professionals [17, 26]. A study conducted on 55 CPs in Jordan utilizing SPM, revealed that older pharmacists with extensive years of experience were more likely to provide necessary information proactively, without awaiting patient inquiries. In contrast, pharmacists with less than five years of experience and higher confidence levels were more inclined to respond when patients specifically sought information. Furthermore, research indicates that as more time elapses since graduation, the knowledge and skills of healthcare professionals may diminish [21, 26–28]. This gap emphasizes the critical need for ongoing education in performing EBM and maintaining clinical expertise.

It is worth mentioning that in a systematic review of 57 articles on physicians’ KAP toward EBM, consulting with colleagues was recognized as the primary source of information for EBM. Interestingly, e-health services were reported as the least favored resource among physicians in this study [29]. In contrast, a study in the United States involving clinical assistants found that over 70% of participants preferred using online resources, indicating a potential shift towards the adoption of e-health platforms in clinical settings [30].

Previous studies have identified the primary resources that pharmacists usually rely on for finding clinical evidence, including international guidelines, textbooks, Wikipedia, Google Scholar, PubMed/Medline, Medscape^®^, The Database of Abstracts of Reviews of Effectiveness (Dare^®^), Cochrane^®^, EvidenceUpdates^®^, the Turning Research into Practice (Trip^®^), LexiComp^®^, Micromedex^®^, and UpToDate^®^ [21, 22, 31]. The reliance on a diverse range of sources highlights the necessity for comprehensive training in the utilization of these tools. Furthermore, it implies that the accessibility to and familiarity with these resources may vary among pharmacists [28, 32, 33].

Recent research has recognized several barriers to the integration of EBM in routine practice. These barriers include time constraints, insufficient knowledge and skills, patient overload, chaotic work environments, lack of financial resources, inadequate facilities to ensure patient privacy, absence of an integrated communication framework within the healthcare system, incomplete access to patient records, insufficient societal awareness regarding the critical role of healthcare professionals, a focus on increasing product sales, inadequate motivation for time-consuming consultations, and restricted access to e-health platforms for pharmacist. Additionally, few articles have highlighted that a primary obstacle to utilizing scientific resources in daily practice is the lack of sufficient authority to modify patient care protocols and resistance to change [2, 14, 31, 34].

This study encountered several obstacles in the collection and reporting of relevant information that could potentially impact the results. The major challenges were the absence of pharmacists in certain pharmacies during the visits, an uneven distribution of community pharmacies across Tehran, patient overload, and difficulties in scheduling meetings with pharmacists.

It is noteworthy that 63.5% of the participants were > 40 years old, and our sample size was limited. Consequently, it can be inferred that in a larger population, the actual level of knowledge and performance may be lower than what was reported in this study. Furthermore, while our cluster-stratified design ensured geographic representation, the reliance on convenience sampling for final pharmacy selection within clusters may introduce a selection bias. This is a common trade-off in community health services research and may limit the generalizability of findings to all community pharmacies within each specific sub-region.

An important consideration is that since participants were required to be clearly informed of the study’s objectives, the SP’s visits were scheduled over a one-week period following their responses to the questionnaires. This timing raises the possibility that pharmacists may have performed better solely during this specific timeframe, potentially influencing our results. Furthermore, as is common in SPM, assessments were conducted by a single trained rater to preserve the naturalistic interaction, a design choice that precluded independent verification, such as using multiple SPs. As mentioned earlier, a standardized binary (yes/no) checklist was specifically designed for the SP to maximize objectivity. This tool functioned as a structured data collection instrument rather than making subjective evaluations. The rigorous training and this structured approach were employed to mitigate the potential for measurement bias inherent in the single-rater design. Future studies should consider implementing a longer observation period, randomizing the scheduling of visits, and employing different SPs simultaneously to better assess the pharmacists’ knowledge and performance across varying conditions. In addition, incorporating a diverse range of assessment methods could provide a more comprehensive evaluation of pharmacists’ competencies and help identify specific areas for improvement.

Given the current challenges within Iran’s pharmaceutical system, many pharmacists may not follow EBM in their daily practice or may not be sufficiently familiar with the tools that support this approach. Our study showed that the use of electronic information databases such as UpToDate^®^ was associated with higher knowledge scores and more evidence-based decision-making among pharmacists. This finding implies that utilizing these valuable resources can bridge the gap between the attitude and actual implementation of EBM in community pharmacies. In this case, the results of our study could alert health policymakers to consider clearer frameworks to enhance the knowledge and performance levels in critical aspects of the health, such as medication consumption during pregnancy, and to address existing barriers to ultimately improve patient quality of life. Further studies are warranted to explore the KAP of Iranian CPs regarding the impact of scientific resources and e-health tools in clinical appraisal by using more standardized questionnaires and more rigorous methodologies.

## Conclusion

In conclusion, our study has revealed that although pharmacists have a positive attitude towards incorporating EBM into their professional practice, there is still a substantial potential and scope for improvement in their knowledge and practice. The results of this study demonstrated a significant association between the use of UpToDate^®^ and the knowledge and practice scores of CPs. Moreover, a positive correlation was observed between higher levels of knowledge and improved practice among pharmacists.

## Supplementary Information

Below is the link to the electronic supplementary material.

Supplementary Material 1

Supplementary Material 2

## Data Availability

All data analyzed during this study is provided within the manuscript.
