# Supplementary material for: Exploration of utilizing electronic health databases in evidence-based practice among Iranian pharmacists: a survey by simulation of health-seeking pregnant women
Source: J Pharm Health Care Sci. 2026 Apr 2;12:40. doi: 10.1186/s40780-026-00561-7 (PMC13047831; doi:10.1186/s40780-026-00561-7)
Supplement: Supplementary file 1 — Supplementary Material 1 [file 40780_2026_561_MOESM1_ESM.docx]

**Knowledge and Attitude Questionnaire**

Please complete the following questionnaire. This will help the project monitor changes in the knowledge, attitudes, and practices of stakeholders regarding data collection and sharing systems.

When selecting an option, please mark the blank box. Please respond to gaps with a clear written statement.

**Demographic characteristics**

1. **Age:**
2. 20-29 □
3. 30-39 □
4. 40-49 □
5. 50-65 □
6. **Gender:**
7. Male □
8. Female □
9. **Highest level of education:**
10. PharmD □
11. PhD □
12. BPharm □
13. **Work experience:**
14. Less than 5 years □
15. 5-10 years □
16. More than 10 years □
17. **Average hours per day working in the pharmacy:**
18. 0-4 hours □
19. 4-8 hours □
20. 8-12 hours □
21. More than 12 hours □
22. **Average hours per month working in the pharmacy:** .......... hours
23. **Pharmacy location in Tehran municipality district:** ..........
24. **Current pharmacy role:**
25. Owner □
26. Technical Assistant □
27. Deputy Technical Assistant □
28. **Years since graduation:**
29. 1-10 years □
30. 11-20 years □
31. 20-30 years □
32. More than 30 years □
33. **University attended:** ..........
34. **Types of nearby practices:**
35. General/family medicine □
36. Specialists (list specialties): □
37. **Work internet access: Yes □ No □**

**Knowledge**

For each question, check "Yes" or "No". If you answer "Yes", briefly answer the subsequent follow-up question in the space provided.

| **Questions** | **Yes** | **No** | **Explanation if Yes** |
| --- | --- | --- | --- |
| 1. Are you familiar with the concept of *P*-value?  - What is its practical significance? |  |  |  |
| 1. Are you familiar with the concept of Odds Ratio?  - What is its practical significance? |  |  |  |
| 1. Are you familiar with the Cochrane Collaboration?  - What types of studies are conducted there? |  |  |  |
| 1. Are you familiar with meta-analyses?  - What is their primary characteristic? |  |  |  |
| 1. Are you familiar with cohort studies?  - What is their primary characteristic? |  |  |  |
| 1. Are you familiar with randomization in clinical trials?  - What is its practical significance? |  |  |  |
| 1. Are you familiar with pharmaceutical/medical resources?  - Please name three of them. |  |  |  |
| 1. Are you familiar with evidence-based medicine?  - What is its practical significance? |  |  |  |

| 1. Rank the following by level of evidence regarding treatment efficacy (1=highest, 6=lowest): |
| --- |
| Case-Series Study ..........  Case Report ..........  Randomized Blinded Controlled Trial ..........  Randomized Open-Label Controlled Trial ..........  Expert opinion ..........  Meta-analysis .......... |

| **Select the best response.** |
| --- |
| 1. What type of study provides the most reliable evidence about risk factors for lung cancer? 2. Cohort Study 3. Prospective Case-Control Study 4. Double-Blinded Placebo-Controlled Study 5. Cross-Sectional Observational Study 6. No opinion. |
| 1. A study compared Drug A, Drug B, and a control. Drug A (p=0.05) and Drug B (p=0.002) were significantly more effective than the control. Which conclusion about Drug A vs Drug B is most valid? 2. Drug A is more effective than drug B. 3. Drug B is more effective than drug A. 4. Not possible to determine effectiveness difference. 5. The two drugs are similarly effective. 6. No opinion. |
| 1. Which resource is best for information on drug incompatibilities? 2. BNF 3. Martindale 4. Symptoms in the Pharmacy 5. Katzung Pharmacology |

**Attitude**

| **Please indicate your level of agreement with the following statements.** | **Agree** | **Disagree** | **Neutral** |
| --- | --- | --- | --- |
| 1. References should be used to answer patient questions. |  |  |  |
| 1. Pharmacist knowledge and experience alone are sufficient. |  |  |  |
| 1. Too busy during work to search for answers for patients. |  |  |  |
| 1. Patients doubt pharmacists' knowledge if we use resources. |  |  |  |
| 1. On-the-job experience is as good as using resources. |  |  |  |
| 1. Resources to check medication safety in pregnancy/breastfeeding should be used for all patients. |  |  |  |
| 1. Pharmacists don't need to check interactions if physicians assess. |  |  |  |
| 1. Providing more medication information causes patient anxiety/confusion. |  |  |  |
| 1. Using reliable resources improves patient satisfaction. |  |  |  |

**General Information**

1. When do you refer to scientific resources to answer patient questions? For each, list the source(s) used:

□ New drug information: ..........

□ Medication use in pregnancy/breastfeeding: ..........

□ Medications for older patients: ..........

□ Medications for infants/children: ..........

□ Checking drug interactions: ..........

□ Identifying drug side effects: ..........

□ Injectable drug incompatibilities: ..........

1. Rate your English proficiency out of 100.
2. Writing: ..........
3. Speaking: ..........
4. Listening: ..........
5. Reading: ..........
6. Which method(s) do you use to search for answers to patient questions? Select all that apply:
7. Google search in Persian
8. Google search in English
9. UpToDate® or similar evidence-based resources
10. I would call my colleagues and professors.
11. Contact colleagues/professors
12. Contact drug information centers
13. Refer patients to drug information centers

**Pharmacy Practice Assessment by Simulated Patient**

| **Patient Care Question** | **Yes** | **No** |
| --- | --- | --- |
| 1. Provided clinical management plan after history? |  |  |
| 1. Noticed the patient's opening statement? |  |  |
| 1. Asked patient's age? |  |  |
| 1. Obtained medical history? |  |  |
| 1. Asked about pregnancy history? |  |  |
| 1. Obtained family health history? |  |  |
| 1. Reviewed current medications? |  |  |
| 1. Assessed for drug or food allergies? |  |  |
| 1. Asked about previous treatments tried? |  |  |
| 1. Documented notes during history-taking? |  |  |
| 1. Paid sufficient attention to symptoms? |  |  |
| 1. Made appropriate recommendations? |  |  |
| 1. Was the patient provided treatment guidance supported by evidence?  - The pharmacist's advice should be verified using reputable resources. |  |  |
| 1. Efficiently selected and appropriately utilized scientific information resources? |  |  |
| 1. Made an appropriate clinical decision? |  |  |

| 1. Professional conduct in patient consultation: 2. Pharmacist wears a professional attire and not a casual clothing 3. Pharmacist attires a clean and tidy coat and not an advertising one 4. Pharmacist introduces themselves and has visible identification 5. Consultation lasts more than 5 minutes 6. Pharmacist follows a structured history-taking approach 7. Pharmacist communicates with confidence to gain the patient's trust 8. Pharmacist possesses the capability to persuade the clients through professional behavior 9. Pharmacist uses respectful language |
| --- |
|  |
|  |
|  |
|  |
|  |
|  |
|  |
|  |
| 1. Education provided in clear, understandable language: 2. Information expressed simply and clearly 3. Avoidance of complex terminology 4. Focus on relevant information 5. Prioritization of key points 6. Repetition of important details 7. Answering additional questions at the end |
|  |
|  |
|  |
|  |
|  |
|  |

| **Final pharmacist statements to patient** | | | **Yes** | **No** |
| --- | --- | --- | --- | --- |
| 1. Provides follow-up recommendations? | | |  |  |
| 1. Requests feedback on consultation? | | |  |  |
| 1. Overall assessment of practice? | Very Poor | Poor | Good | Excellent |
