# Supplementary material for: Exploration of utilizing electronic health databases in evidence-based practice among Iranian pharmacists: a survey by simulation of health-seeking pregnant women
Source: J Pharm Health Care Sci. 2026 Apr 2;12:40. doi: 10.1186/s40780-026-00561-7 (PMC13047831; doi:10.1186/s40780-026-00561-7)
Supplement: Supplementary file 2 — Supplementary Material 2 [file 40780_2026_561_MOESM2_ESM.docx]

The following table is the scoring system for the knowledge questionnaire.

| **Categories of Questions** | | **Expert Panel** | | | | | | | | | | **Average** |
| --- | --- | --- | --- | --- | --- | --- | --- | --- | --- | --- | --- | --- |
|  |  | 1 | 2 | 3 | 4 | 5 | 6 | 7 | 8 | 9 | 10 |  |
|  |  | **Scores** | | | | | | | | | |  |
| 1 | Awareness of the parameters for evaluating clinical studies | 5 | 5 | 10 | 5 | 5 | 1 | 10 | 10 | 3 | 5 | **6** |
| 2 | Awareness of the hierarchy of evidence in clinical studies | 5 | 5 | 5 | 5 | 5 | 2 | 15 | 5 | 3 | 5 | **6** |
| 3 | Awareness of the application of EBM in clinical judgment | 20 | 25 | 30 | 20 | 20 | 25 | 25 | 20 | 13 | 15 | **21** |
| 4 | Familiarity with information sources | 30 | 25 | 20 | 25 | 30 | 30 | 20 | 15 | 20 | 20 | **24** |
| 5 | Awareness of the value of each information source | 15 | 15 | 10 | 15 | 15 | 20 | 10 | 15 | 15 | 15 | **15** |
| 6 | Awareness of the databases for validating clinical studies | 15 | 20 | 20 | 15 | 15 | 15 | 5 | 10 | 30 | 20 | **17** |
| 7 | Awareness of sensitivity assessment indicators | 5 | 2.5 | 2.5 | 5 | 5 | 2 | 5 | 10 | 6 | 10 | **5** |
| 8 | Awareness of methods for evaluating the results of articles and assessing their reliability | 5 | 2.5 | 2.5 | 10 | 5 | 5 | 10 | 15 | 10 | 10 | **8** |
| **Total** | | 100 | 100 | 100 | 100 | 100 | 100 | 100 | 100 | 100 | 100 | **100** |

The following table is the scoring system for the practice assessment. If the participant did not inquire about a particular question, a score of zero was assigned for that response.

| **Number of Question** | **Scores** | **Coefficient** |
| --- | --- | --- |
| 1 | 2 | 3 |
| 2 | 2 | 1 |
| 3 | 2 | 1 |
| 4 | 2 | 1 |
| 5 | 2 | 2 |
| 6 | 1 | 0.5 |
| 7 | 1-3 (hint: 1 point for each question) | 2 |
| 8 | 1 | 0.5 |
| 9 | 1-3 (hint: 1 point for each question) | 1 |
| 10 | 3 | 1 |
| 11 | 1-2 (hint: 1 point for inadequate response, 2 points for adequate response) | 3 |
| 12 | 1-3 (hint: 1 point for 1-2 recommendations, 2 points for 3-4 recommendations, 3 points for more than 4 recommendations) | 1 |
| 13 | 2 | 1.5 |
| 14 | 1-2 (hint: 1 point for inadequate response, 2 points for adequate response) | 10 |
| 15 | 1-2 (hint: 1 point for inadequate response, 2 points for adequate response) | 3 |
| 16 | 4 (hint: 0.5 point for each item) | 5 |
| 17 | 3 (hint: 0.5 point for each item) | 3 |
| 18 | 2 | 1 |
| 19 | 2 | 1 |

The formula of total score was:

$$\boldsymbol{Total Score =}\sum\boldsymbol{(Scores \times Coefficient)}$$

The overall assessment of the practice was based on the total score:

- Very poor: 0-25
- Poor: 25-50
- Good: 50-75
- Excellent: 75-100
